# Triple Culture of Primary Human Osteoblasts, Osteoclasts and Osteocytes as an In Vitro Bone Model

**DOI:** 10.3390/ijms22147316

**Published:** 2021-07-07

**Authors:** Anne Bernhardt, Jasmin Skottke, Max von Witzleben, Michael Gelinsky

**Affiliations:** Centre for Translational Bone, Joint- and Soft Tissue Research, Medical Faculty and University Hospital, Technische Universität Dresden, D-01307 Dresden, Germany; jasminskottke@hotmail.de (J.S.); max.von_witzleben@tu-dresden.de (M.v.W.); michael.gelinsky@tu-dresden.de (M.G.)

**Keywords:** osteocyte, osteoclast, osteoblast, co-culture, in vitro, BGLAP, bone model

## Abstract

In vitro evaluation of bone graft materials is generally performed by analyzing the interaction with osteoblasts or osteoblast precursors. In vitro bone models comprising different cell species can give specific first information on the performance of those materials. In the present study, a 3D co-culture model was established comprising primary human osteoblasts, osteoclasts and osteocytes. Osteocytes were differentiated from osteoblasts embedded in collagen gels and were cultivated with osteoblast and osteoclasts seeded in patterns on a porous membrane. This experimental setup allowed paracrine signaling as well as separation of the different cell types for final analysis. After 7 days of co-culture, the three cell species showed their typical morphology and gene expression of typical markers like *ALPL, BSPII, BLGAP, E11, PHEX, MEPE, RANKL, ACP5, CAII* and *CTSK*. Furthermore, relevant enzyme activities for osteoblasts (ALP) and osteoclasts (TRAP, CTSK, CAII) were detected. Osteoclasts in triple culture showed downregulated TRAP (*ACP5*) and *CAII* expression and decreased TRAP activity. *ALP* and *BSPII* expression of osteoblasts in triple culture were upregulated. The expression of the osteocyte marker E11 (*PDPN*) was unchanged; however, osteocalcin (*BGLAP*) expression was considerably downregulated both in osteoblasts and osteocytes in triple cultures compared to the respective single cultures.

## 1. Introduction

Bone is a complex dynamic tissue being permanently remodeled through bone forming osteoblasts and bone resorbing osteoclasts orchestrated by osteocytes. These three cell types are also key players in bone regeneration after trauma, infection or resection. Bone defects lacking physiological healing are increasingly treated with synthetic functional biomaterials as an alternative to auto- or allografts. In most cases, in vitro testing of novel bone graft materials is restricted to biocompatibility analysis using osteoblasts or osteoblast precursors. However, it has become clear, that also osteoclasts [1] and osteocytes [2] play an important role in bone healing and should be considered for in vitro investigations of bone graft materials. Because of this, performing pre-clinical testing of bone graft biomaterials using co-cultures of bone cells has come into focus. Moreover, in vitro co-cultures of bone cells can be important tools to investigate the cross-talk between osteocytes, osteoblasts and osteoclasts, which is important for the understanding of bone metabolism and diseases [3,4,5,6]. Several approaches have been published combining osteoblasts and osteoclasts in different in vitro co-culture systems [7,8]. The intention behind these co-culture approaches is to better understand the interaction of osteoblasts and osteoclasts during bone remodeling or to test drugs and biomaterials/scaffolds in an environment, which is closer to human bone. Bone cell co-cultures comprising osteocytes are studied less comprehensively. The reason for that lies in the limited availability of primary osteocytes, since the isolation of these post-mitotic cells from bone tissue is tedious [9,10,11]. Therefore, co-cultures of osteoclasts and osteocytes were mainly performed with murine osteocytic cell lines [12,13,14] and only two osteoclast/osteocyte co-culture models with primary cells have been reported so far [15,16]. A similar situation applies to co-cultures of osteoblasts and osteocytes, most of them being performed with murine osteocytic cell lines [17,18,19]. We previously performed a first co-culture study involving both primary human osteoblasts and osteocytes [20]. There are only few approaches to co-cultivate more than two bone or bone-related cell species. This is mainly because of the increasing complexity of those systems, as each cell species requires special media composition [21] and environment. Clarke and co-workers combined primary human osteoblasts and osteoclast precursors in a scaffold-free rotational culture which resulted in mineralized constructs comprising the three bone cell species osteoblasts, osteoclasts and osteocytes after 21 days of cultivation [22]. However, the design of this study did not allow separate analysis of the single cell species. Since vascularization is a central requirement for bone regeneration, some studies reported in vitro bone triple cultures including human osteoblasts, osteoclasts and endothelial cells. Pagani and co-workers were able to show that cells in triple co-culture behave differently compared to single cultures of the same cell type and propose this model for future biomaterials testing [23]. Gremare and co-workers established a similar triple culture as a first step to study bone cell communication [24]. Wein et al. proposed a triple culture model of osteoblast precursors, endothelial cells and fibroblasts to evaluate bone implant materials [25].

The aim of the present study was to establish an in vitro triple culture model involving human osteoblasts, osteoclasts and osteocytes in a spatially defined three-dimensional environment allowing both cell–cell interactions of all three cell types and separate analysis of the single-cell species. Proceeding from our experience with co-culture models of primary human osteocytes with osteoclasts [16] and osteoblasts [20], we aimed to develop a more complex system comprising the three major bone cell species. To this end, we used a combination of collagen gels with transwell inserts, to separate gel-embedded osteocytes from the other cell types with a porous membrane as already established for co-culture approaches [16,20]. To separate osteoclasts and osteoblasts, which were seeded on the other side of the porous membrane, we used silicon grids, which were temporarily applied during seeding to establish a patterned distribution of the two cell species. To the best of our knowledge, this is the first report on an in vitro triple culture of human primary osteoblasts, osteoclasts and osteocytes with separate analysis of the three cell species.

## 2. Results

### 2.1. Triple Culture Setup

**Figure 1:**
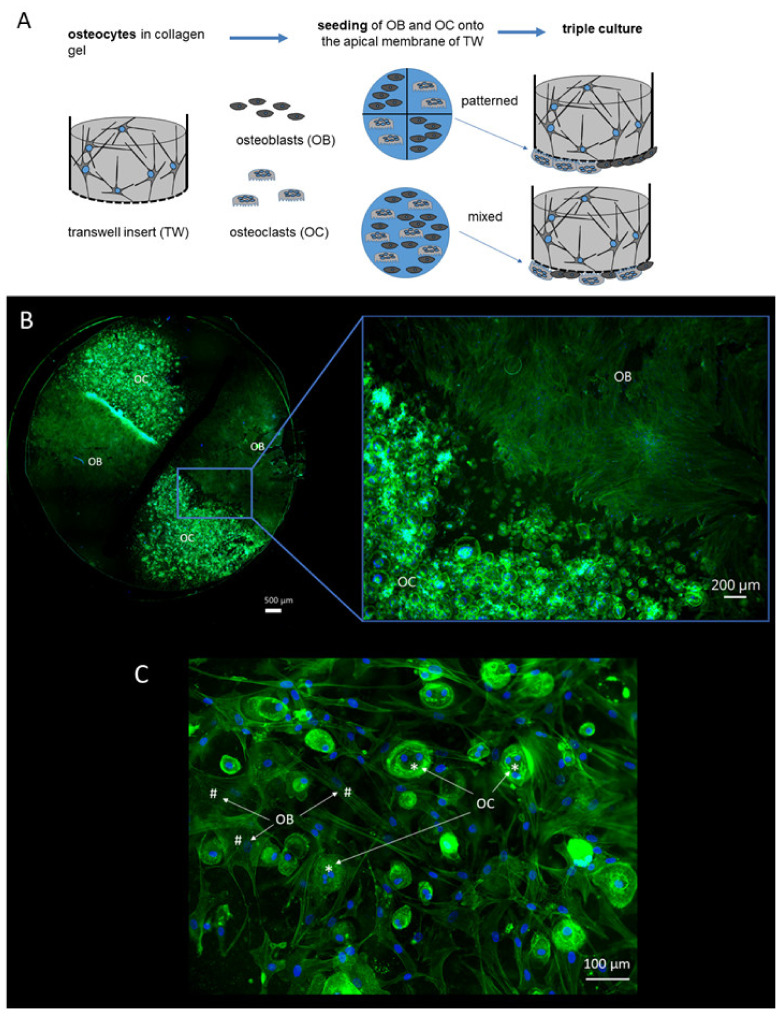
Triple culture setup for patterned and mixed seeding of OC and OB (**A**) OB and OC on the porous membrane after patterned seeding (**B**) as well as mixed seeding (**C**) and 7 days of triple culture. Fixed cells were stained with Alexa fluor 488 phalloidin and DAPI to stain cytoskeleton (green) and nuclei (blue).

Two different setups were used for triple culture of osteoblasts, osteoclasts and osteocytes (Figure 1A). In both setups, osteocytes were differentiated from primary human osteoblasts in collagen gels, located in commercially available transwell inserts under low serum conditions (2% FCS). After osteocytic differentiation for 2 weeks, osteoblasts and osteoclasts were seeded to the apical side of the porous membrane and the triple cultures were maintained for another 7 days again under low serum conditions. In five experiments, silicon grids were used to separate osteoblasts and osteoclasts during seeding (“patterned seeding”). In another four experiments, the cells suspensions containing osteoblasts and osteoclasts were mixed before seeding the membrane (“mixed seeding”). The patterned seeding of mature osteoclasts and osteoblasts (exp. 1–5, see Materials and Methods,) allowed separate analysis of these two cell species. After removing the silicon grid and cultivation for one week, the cell populations were mainly colonized within their allocated areas (Figure 1B). There were only few areas with direct cell–cell contact. Triple cultures, in which osteoblasts and osteoclasts were not seeded separately, showed direct cell–cell contact between osteoblasts and osteoclasts (Figure 1C).

### 2.2. Cell Morphology in Separated Triple Cultures and Single Cultures

**Figure 2:**
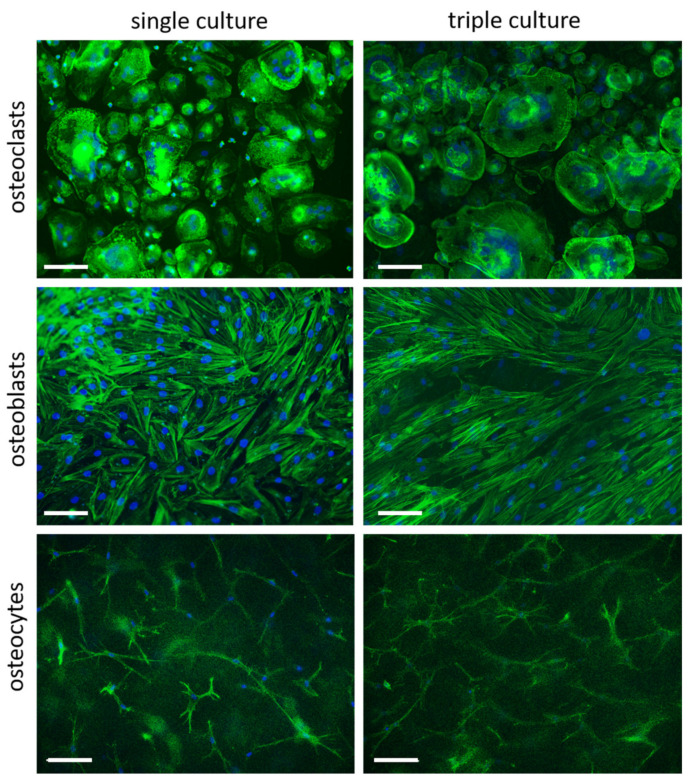
Fluorescence microscopic images of osteoblasts and mature osteoclasts seeded on the porous PET membrane, as well as osteocytes, embedded in collagen I gels after 7 days in single and triple culture, respectively. Scale bars represent 100 µm. Nuclei appear in blue, cytoskeleton in green.

Cell morphology of osteoclasts, osteoblasts and osteocytes in separated triple cultures was not changed compared to the respective single cultures (Figure 2), indicating, that all cell species are able to retain their specific phenotype also in triple culture.

### 2.3. Osteocytes in Triple Culture

**Figure 3:**
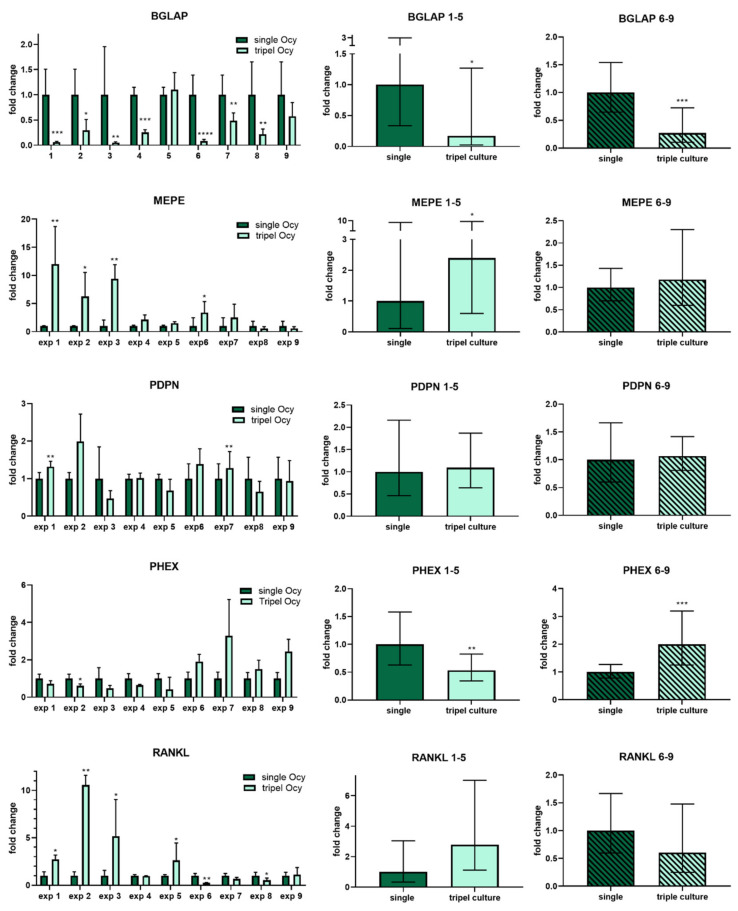
Gene expression analysis of osteocyte marker genes *BGLAP, E11, RANKL, PHEX* and *MEPE* for osteocytes cultivated in single culture compared to triple culture with osteoclasts and osteoblasts. Left column shows data of 9 different experiments. Middle column: Summarized data for experiments 1–5. Osteocytes were co-cultivated with spatially separated osteoblasts and osteoclasts (patterned seeding). Right column: Summarized data for experiments 6–9 (mixed seeding of OB and OC). Gene expression was normalized to the expression of *GAPDH* and fold changes were calculated using the CT method. Fold changes +/− upper and lower limits. * *p* < 0.05; ** *p* < 0.01, *** *p* < 0.001, **** *p* < 0.0001.

Osteocytic marker genes *BGLAP* (osteocalcin), *PDPN* (E11), *RANKL*, *PHEX* and *MEPE* were detected in both single and triple culture of gel-embedded osteocytes. Experiments with patterned seeding of OB and OC (exp. 1–5) showed similar gene expression compared to experiments with mixed seeding (exp. 6–9). Gene expression analysis revealed a significant decrease in osteocalcin mRNA expression for both groups in triple culture, while gene expression of *PDPN* (E11) and *RANKL* was not significantly changed (Figure 3). *PHEX* expression was decreased and *MEPE* expression increased in osteocytes co-cultivated with separated osteoblasts and osteoclasts (exp 1–5), while *PHEX* expression was increased and *MEPE* remained stable in osteocytes co-cultivated with mixed osteoblasts and osteoclasts (exp. 6–9) (Figure 3).

### 2.4. Osteoclasts in Triple Culture

**Figure 4:**
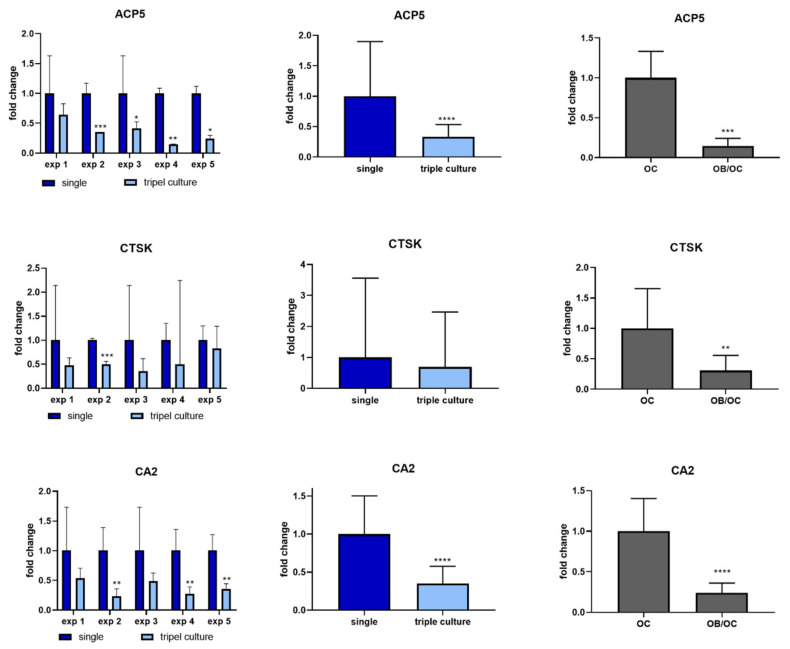
Gene expression analysis of osteoclast marker genes *ACP5, CA2* and *CTSK* for osteoclasts cultivated in single culture compared to triple culture (left and middle column) and in co-culture with osteoblasts (right column). Gene expression was normalized to the expression of *GAPDH* and fold changes were calculated using the CT method. Fold changes +/− upper and lower limits are given for every triple culture experiment (left column) for all five triple culture experiments together (middle column). Additionally, osteoclasts, derived from two different donors were combined with osteoblasts of one donor in two independent experiments in co-culture (right column) * *p* < 0.05; ** *p* < 0.01, *** *p* < 0.001, **** *p* < 0.0001.

**Figure 5:**
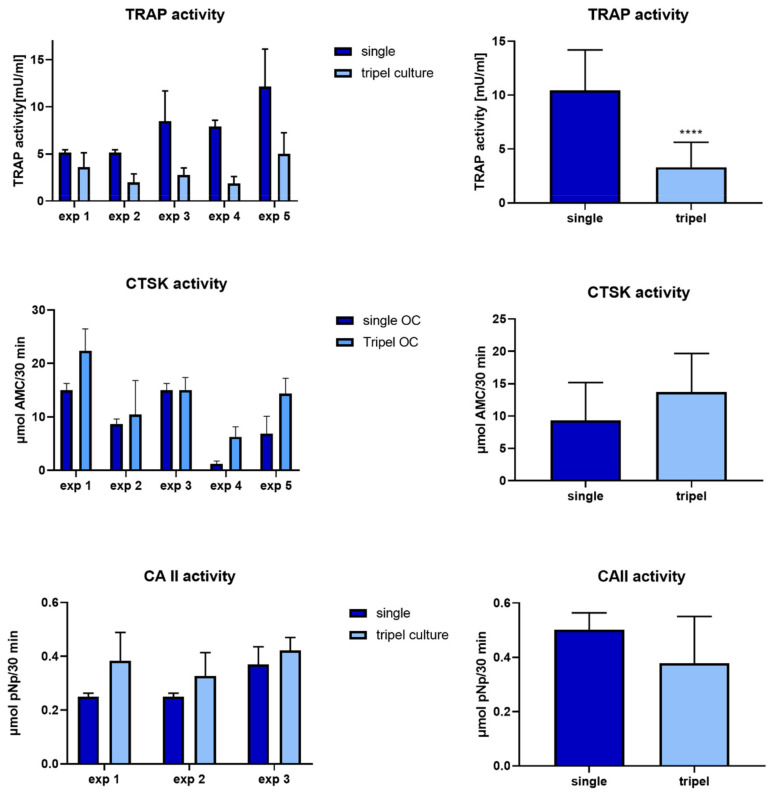
Activities of osteoclast-specific enzymes TRAP, CTSK and CAII for osteoclasts cultivated in single culture compared to triple culture. Average +/− standard deviation for each experiment (left column) and for all five experiments together (right column). **** *p* < 0.0001.

Osteoclast marker expression was analyzed in separated triple cultures compared to single cultures (patterned seeding, exp. 1–5). All examined osteoclast markers, *ACP5* (TRAP), *CTSK* and *CA2* were detected on mRNA level in single and triple culture. Gene expression analysis revealed significantly decreased *ACP5* and *CA2* expression in triple cultures compared to single osteoclast cultures, while CTSK mRNA expression was not changed (Figure 4, left and middle column). Downregulation of *ACP5*, *CTSK* and *CA2* was furthermore detected in additional experiments in co-cultures of osteoclasts and osteoblasts (Figure 4, right column). Osteoclast markers TRAP, CA2 and CTSK were furthermore analyzed on protein level by quantifying their enzymatic activities (Figure 5). TRAP activity was significantly reduced in triple cultures compared to osteoclast single culture, while no significant changes were detected in case of CA2 and CTSK activities (Figure 5).

### 2.5. Osteoblasts in Triple Culture

**Figure 6:**
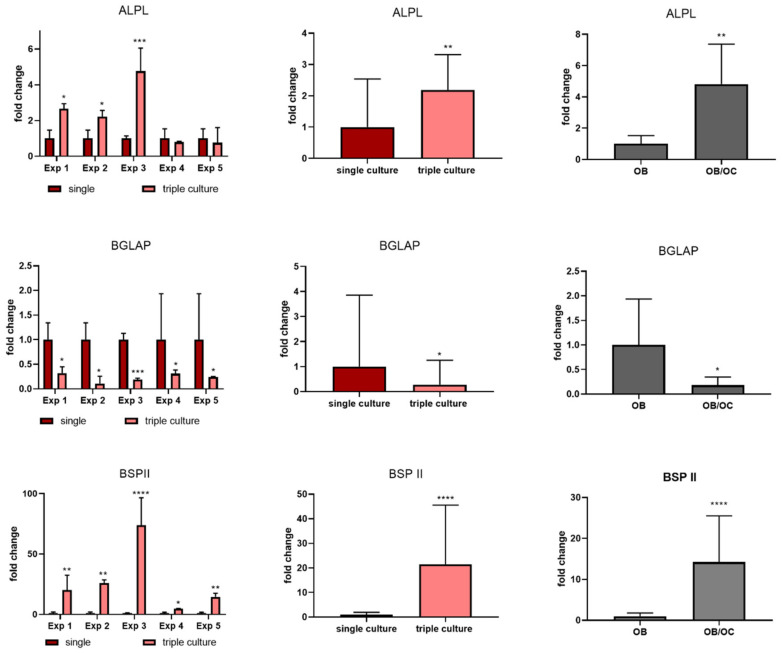
Gene expression analysis of osteoblast marker genes ALPL, BGLAP and BSPII for osteoblasts cultivated in single culture compared to triple culture with osteoclasts and osteocytes. Gene expression was normalized to the expression of GAPDH and fold changes were calculated using the ΔΔCT method. Fold changes +/− upper and lower limits for the single experiments (left column) and for all five experiments together (middle column). Additionally, osteoclasts derived from two different donors were combined with osteoblasts of one donor in two independent experiments in co-culture (right column). * *p* < 0.05; ** *p* < 0.01, *** *p* < 0.001, **** *p* < 0.0001.

**Figure 7:**
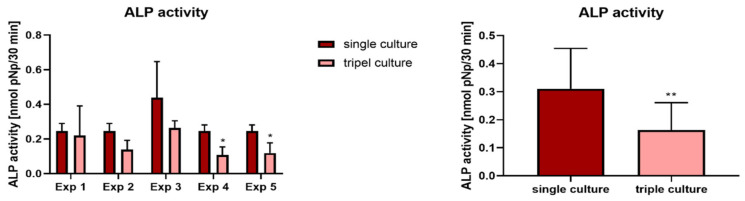
ALP activity of osteoblasts in single culture compared to triple cultures with osteoclasts and osteocytes. Average +/− standard deviation for every experiment (**left**) and for all five experiments together (**right**). * *p* < 0.05, ** *p* < 0.01.

Gene expression analysis showed distinct differences between single and triple cultures: Osteoblast marker genes *ALPL* and *BSP II* were significantly upregulated in triple culture, while *BGLAP* (osteocalcin) was significantly downregulated (Figure 6, left and middle column). Especially for *BSPII* the effect was highly significant. However, similar effects were also detected in additional co-cultures of osteoclasts and osteoblasts under the same experimental conditions (Figure 6, right column). Interestingly, ALP activity was reduced in triple culture, which is in contrast to the upregulated *ALPL* mRNA upregulation (Figure 7).

## 3. Discussion

Bone metabolism and development are based on the concerted action of osteoblasts and osteoclasts, orchestrated by matrix-embedded osteocytes. The complex regulation of differentiation and activity of osteoblasts and osteoclasts is achieved by molecular signaling between bone cells, involving a network of receptors, cytokines, growth and transcription factors, cell-specific enzymes and ligands [26]. Signaling pathways between bone cells have been comprehensively studied in animal models. In vitro bone models, however, are challenging to create, due to the variety of bone tissue functions and the complex interaction between bone cells. Therefore, current models focus on certain aspects of bone biology like recreating the bone marrow niche, modelling vasculogenesis or generating tumor models [27]. The focus of the present study was to establish a model, which can be applied for the evaluation of the effect of bioactive molecules, drugs and biomaterials extracts on bone cell differentiation and interaction. The aim was to co-cultivate the three major bone cell species under conditions allowing both paracrine signaling of the cells and separate analysis. Those conditions could also be realized by organ-on-a-chip approaches. A few promising studies have been published in the field of bone-on-a chip studies. The group of Marnie Saunders has developed a microfluidic system, which allows the analysis of osteoblasts or osteoclasts in response to osteocyte-conditioned medium [28,29]. Middleton et al. co-cultivated osteocytes and osteoclasts in a microfluidic system and demonstrated an increased mechano-response of osteocytes in co-culture with osteoclasts [30]. Sun and co-workers were the first to use primary osteocytes instead of osteocyte cell lines for a bone-on-a chip study, however, they did not co-cultivate the cells with other bone cell species [31]. Microfluidic devices allow controlled cultivation of cells and the application of perfusion and mechanical loading; however, perfusion can also hamper paracrine signaling due to the higher volume of medium used. Furthermore, microfluidic devices are quite complex and therefore do not allow the preparation of high sample numbers, which are a prerequisite for statistically sound results, especially when primary cells of different donors are used. In the present study, therefore, simple devices—commercially available cell culture inserts—were applied to establish bone triple cultures. The main component of the model is a collagen gel which allows the cultivation of osteocytes in a 3D environment, which is important to support osteocytic differentiation and support of osteocytic phenotype [20,32,33,34]. The porous membrane of the cell culture insert was used for two reasons, (1) to prevent migration of osteoblasts seeded on top of the gel [35] and (2) to allow separate RNA isolation from the different cell species. This separation of cell species might be associated with limited cell–cell contact. However, Honma and co-workers demonstrated the ability of mouse osteocytes to stimulate osteoclast formation efficiently from the opposite side of a porous membrane. It was shown that osteocytic RANKL was provided as membrane-bound form through osteocyte dendritic processes [15]. In physiological bone, osteocytes are deeply embedded in the matrix, while osteoblasts and osteoclasts reside more on the surface of the bone, a spatial arrangement, which is roughly mimicked in the proposed triple culture model. With respect to this, a limitation of the proposed model is that osteoblasts and osteoclasts are cultivated on non-resorbable membranes. However, coating of the porous membranes with a mineral phase or extracellular bone matrix would hamper the exchange of signaling molecules between osteocytes and the other bone cells and was therefore not applied. Further research should focus on replacing the porous PET membrane by a physiological porous material.

The complexity of in vitro models increases with the number of involved cells due to the different requirements of medium composition [36,37]. In the proposed triple culture model, media requirements of osteoclasts and osteocytes are contradictory, since 10% serum supplementation is necessary to allow osteoclastic differentiation [38], while osteocytes require serum concentrations as low as 0.2% FCS [39] and 2.5% FCS [9] to maintain their differentiation state. We already faced this problem when co-cultures of osteocytes and osteoclasts were established. To circumvent the different media requirements, the formation of osteoclasts was performed separately and mature osteoclasts were transferred to the co-cultures, which were further cultivated in the presence of 2% FCS [16]. For the addition of osteoblasts as the third cell species, we developed silicon grids, which stuck to the porous membrane and separated osteoblasts and osteoclasts. The grids allowed a separate application of different cell suspensions and were removed after the cells had attached to the membrane.

In the established triple cultures, all cell species showed their typical morphology and there were no obvious morphological differences between single and triple cultures. The good balance between the three cell species is a prerequisite to use those triple cultures in future to investigate the influence of bioactive molecules, drugs and biomaterial extracts. As expected, due to the signaling between the cells we detected differences between single and triple cultures on mRNA level.

Osteoclasts in triple culture showed a significantly decreased mRNA expression of TRAP (*ACP5*) and *CA2*, accompanied by a significant decrease of TRAP activity. In our previous study, where osteoclasts and osteocytes were co-cultivated, we did not find such significant changes of TRAP mRNA expression and activity [20], indicating, that the presence of osteoblasts in the triple culture setup is responsible for these changes. This was confirmed by co-culture experiments of osteoblasts and mature osteoclasts on transwell membranes and cell-free collagen gels on the other side of the membrane. The presence of osteoblasts is sufficient to downregulate osteoclast-specific genes *ACP5*, *CA2* and *CTSK* significantly. It has been shown before that TRAP activity of human osteoclasts is significantly reduced when the cells are cultivated in indirect co-culture with osteogenically differentiated MSC [40,41]. On the other hand, osteoblasts and osteocytes are the major source of RANKL [42], which is the key molecule in the regulation of osteoclast differentiation and function and, therefore, co-cultivation with osteoblasts/osteocytes would be expected to increase osteoclast formation and activity. Accordingly, Steller and co-workers demonstrated a significantly increased osteoclast number in co-cultures with osteoblasts [43]. In contrast to other studies, the osteoclasts in our study are already differentiated when introduced to the triple culture. Therefore, the supplementation with RANKL, which is produced by the osteocytes and osteoblasts in triple culture, does not further increase the expression of osteoclast-specific genes. As already pointed out, the decrease in osteoclast marker expression was not accompanied by a reduction of osteoclast number or changes in osteoclast morphology.

Osteoblasts, which were cultivated in triple culture, displayed a significantly increased mRNA expression of the osteogenic markers *ALPL* and *BSP II* compared to single cultures. In our previous study on co-cultures of osteoblasts and osteocytes, the *BSPII* expression of osteoblasts was also increased, but only for one of the two examined osteoblast donors [15]. Additional experiments with co-cultures of osteoclasts and osteoblasts in the very same setup, which was used for the triple cultures (transwell inserts, silicon grids for separation), also revealed significant upregulation of *ALPL* and *BSPII* in osteoblasts, indicating that both the presence of osteoclasts and osteocytes might be responsible for the increased *ALPL* and *BSPII* expression in the triple culture. It has been reported before that osteoclasts also express BSPII [44]. However, in our experiments we only found marginal *BSP II* expression (CT values around 39) in single cultures of osteoclasts, while osteoblasts both in single and triple culture showed high *BSPII* expression. BSP II is associated with the maturation of osteoblasts and with the formation of extracellular bone matrix [45]. It has been shown before that osteoclasts secrete the so-called “coupling factors” such as collagen triple helix repeat containing 1 (CTHRC1), complement component c (C3) or sphingosin phosphate kinase [46], as well as cardiotrophin_1 [47], which directly or indirectly stimulate osteoblast differentiation. Accordingly, a stimulation of osteogenic differentiation of mesenchymal stem cells in direct or indirect co-culture with osteoclasts was reported before [48,49]. The upregulation of osteoblast markers ALP and BSP II in the present study can therefore be contributed to the presence of osteoclasts in the triple cultures. In contrast, osteocalcin (*BGLAP*) expression of osteoblasts was significantly decreased in triple cultures of the present study, as well as in co-cultures with osteoclasts in the additional co-culture experiments. However, when osteoblasts were cultivated in the presence of osteocytes only, gene expression of *BGLAP* was significantly increased [15]. Our hypothesis on this is that osteocytes and osteoclasts may have contradictory effects on *BGLAP* expression of osteoblasts. While osteocytes apparently stimulate *BGLAP* expression in osteoblasts, osteoclasts seem to inhibit BGLAP expression in osteoblasts. Looking into the spatial arrangement of the cells in triple culture, the exchange of molecules seems to be facilitated between osteoblasts and osteoclasts, since both are colonized on the same side of the PET membrane, while osteocytes are separated from the other cell species by both the porous membrane and the collagen gel. This might provide an explanation for the stronger effect of osteoclasts compared to osteocytes on osteocalcin expression in osteoblasts.

Osteocytes have been shown to exert numerous effects on both osteoblasts and osteoclasts, orchestrating the balance between bone formation and resorption [2]. In the present study, we were looking additionally for a possible effect of osteoblasts and osteoclasts on osteocyte marker expression. The expression of osteocyte marker genes *PHEX*, *MEPE*, *RANKL* and *E11* (*PDPN*) remained nearly unchanged in the overall view of all nine experiments, even if there were some significant changes in the single experiments. *PHEX* expression was significantly downregulated in triple cultures with patterned seeding of osteoblasts and osteoclasts, while it was significantly upregulated in triple cultures of osteocytes with a mixed population of osteoblasts and osteoclasts. PHEX is an enzyme which plays a role in the regulation of phosphate homeostasis and extracellular matrix mineralization [50] and its expression increases greatly during the transition of osteoblasts to osteocytes [51]. Our data suggest that the presence of osteoblasts and osteoclasts with low cell–cell contact does not support PHEX expression in co-cultured osteocytes, while mixed osteoclast/osteoclast cultures do. However, the experiments were further conducted with different osteoblast/osteocyte donors, so the observed effect can also be caused by donor variations. In previously conducted co-culture studies: both co-cultures of osteocytes with osteoclasts [16] and osteoblasts [20] did not show significant effects on osteocyte marker expression. Prideaux and co-workers reported a stimulating effect of osteoblast matrix mineralization on E11 expression in osteocytes [52]. In the present experimental setup, such a stimulating effect was not observed. Possibly, the different experimental setup of the study (cell lines versus primary cells, different co-culture setup) led to these contradictory results. Furthermore, we did not check, whether the osteoblasts in triple culture mineralized their matrix. The relatively short co-cultivation time of 7 days as well as the low serum concentration might prevent matrix mineralization of osteoblasts, which will be part of further investigations. Interestingly, *BGLAP* expression of osteocytes was significantly downregulated in triple cultures compared to single cultures of osteocytes. Osteocalcin (*BGLAP*) is an early marker of osteocytes, and a late marker of osteoblasts. Apparently, in both osteoblasts and osteocytes its gene expression is downregulated in the presence of mature osteoclast, and there were no differences between mixed and patterned seeding of osteoclasts and osteoblasts. This allowed us to compare osteocyte gene expression data of nine different experiments with high statistic relevance. Comparing the results of nine different experiments, *BGLAP* expression of osteocytes in triple culture was significantly decreased, while there was no significant effect on other osteocyte markers in this overall view. Considering these results, it becomes obvious, that in triple cultures *BGLAP* expression is downregulated in both osteocytes and osteoblasts. This is in contrast to the observed stimulating effect on osteoblastic differentiation, which became incident by the increased *ALPL* and *BSPII* expression of osteoblasts. Further studies will include the detection of osteocalcin on protein level in single and triple cultures, and studies on the impact of different factors like Ca2+ [53] and vitamin D3 [54] on osteocalcin gene and protein expression in triple cultures.

The application of solely primary human cells in this model has the advantage of being close to the clinical situation, however, it should also be considered, that this experimental setup requires a large number of samples to get statistically secured data despite donor variances of the cells. Therefore, the proposed triple culture model is time-consuming and expensive and might be not suitable for routine evaluation of biomaterials. Nevertheless, to the best of our knowledge, the proposed model is the first in vitro bone model comprising three primary human bone cell species and can help to answer specific bone-related research questions.

## 4. Materials and Methods

### 4.1. Cell Isolation and Differentiation

Human pre-osteoblasts were isolated from human femoral heads of osteoarthritic patients undergoing total hip replacement at the University Hospital Carl Gustav Carus Dresden (Germany) after informed consent (approval by the ethics commission of TU Dresden). Material of three donors was used (Table 1). Spongious bone fragments (1–2 mm) were digested by two collagenase II treatments as previously described [11]. Cells were expanded in α-MEM with glutamax (Gibco), 15% FCS (Corning), 100 U/mL penicillin and 100 µg/mL streptomycin (PS) (Gibco) until passage 3 before being differentiated into osteoblasts and osteocytes.

#### 4.1.1. Osteoblasts

For differentiation into osteoblasts, pre-osteoblasts were cultivated for 7–9 days in osteogenic medium (α-MEM with glutamax containing 10% FCS, PS, 10−7 M dexamethasone, 10 mM β-glycerophosphate and 12.5 µg/mL ascorbic acid-2-phosphate, all osteogenic supplements were from Sigma-Aldrich, St. Louis, MO, USA). Cells were harvested using Trypsin/EDTA.

#### 4.1.2. Osteocytes

Human osteoblasts, after differentiation in osteogenic medium as described above, were mixed with 8 parts of collagen solution (4 mg/mL rat tail collagen, Meidrix Biomedicals Esslingen, Germany), one part of 10x HBSS, 10 mM β-glycerophosphate, 12.5 mM ascorbic acid-2-phosphate, neutralized with 1 N NaOH in a final concentration of 8 × 104 cells/mL. Each 500 µL of this collagen/cell suspension was pipetted to 12-well transwell inserts with 0.4 µm pore size (Sarstedt, Nümbrecht, Germany) and allowed to gel in the incubator for 30 min. After the collagen gels had formed, α-MEM with glutamax containing 2% FCS, PS, 10 mM β-glycerophosphate and 12.5 µg/mL ascorbic acid-2-phosphate was added (osteocyte differentiation medium, 500 µL to the transwell insert and 2 mL to the well plate) and the constructs were incubated for 14 days for the differentiation of osteocytes from osteoblasts as described previously [20]. Medium was changed after 7 days of cultivation.

#### 4.1.3. Osteoclasts

Peripheral blood mononuclear cells (PBMC) as osteoclast precursors were isolated from leucocyte concentrates (*buffy coats*) of four different blood donors (purchased from German Red Cross, Dresden) by density gradient centrifugation, followed by lysing of erythrocytes as previously described [55]. Osteoclast precursors were differentiated to mature osteoclasts as previously described [56]. In short, PBMC, containing 1 × 107 monocytes were seeded to 25 cm2 ultra-low attachment cell culture flasks (Corning, Corning, NY, USA) in α-MEM with glutamax containing 10% heat-inactivated FCS and PS. After one day, medium was changed to α-MEM with glutamax, 5% heat-inactivated FCS, 5 % human AB serum and PS supplemented with 25 ng/mL MCSF and 50 ng/mL RANKL (both from PeproTech, Rocky Hill, NJ, USA). Cells were cultivated for further 5 days with one medium change in between. After formation of multinucleated cells, the medium was aspirated, the cell layer was washed with PBS and the flasks were shaken with 2 mM EDTA and 0.5% BSA in PBS for 20 min at room temperature to detach the osteoclasts. Cell suspension was centrifuged, the pellet was washed with PBS and cells were resuspended in medium for seeding.

### 4.2. Triple Cultures of Osteocytes, Osteoblasts and Osteoclasts

For the spatial partitioning of the cell types, a silicone grid was produced using a commercial 3D printer (GeSiM, Radeberg, Germany). For this purpose, commercially available silicone was printed in several layers using a needle with an internal diameter of 410 µm, a pressure of 110 kPa and a printing speed of 10 mm/s. Subsequently, the silicone was left to cure for at least 24 h. Grids were disinfected with 70% ethanol for 20 min before use in cell culture. After disinfection, the grids were washed twice with PBS.

Triple cultures of osteocytes, osteoblasts and osteoclasts with separated seeding of osteoclasts and osteoblasts were conducted in five independent experiments (exp. 6–9 involving cells of two osteocyte/osteoblast donors and PBMC of four different donors (exp. 1–5, Table 1). Additionally, four independent experiments were conducted with mixed seeding of osteoblasts and osteoclasts, involving two donors of osteocytes/osteoblasts and PBMC (exp. 6–9, Table 1). For each experiment, single cultures of osteocytes (embedded in collagen gels; exp. 1–9), osteoclasts and osteoblasts (only for exp. 1–5) were carried along with the same conditions of the triple culture concerning cell number and medium.

**Figure 8:**
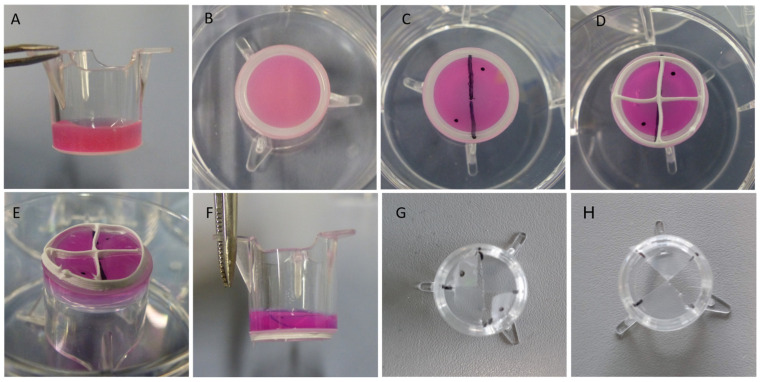
Experimental setup for the triple cultures of osteocytes, osteoclasts and osteoblasts. (**A**) For osteocyte maturation, human primary osteoblasts were embedded in neutralized collagen solution, which was allowed to gel in transwell inserts and cultivated for 14 days in the presence of osteogenic supplements in 12-well tissue culture plates. (**B**) The constructs were removed from the culture dishes and placed bottom-up into 6-well tissue culture plates. (**C**) The PET membrane of the inserts was marked with a sterile pen. (**D**) Silicon grids were placed to the basolateral side of the membrane. (**E**) Cell suspensions of osteoblasts and mature osteoclasts were pipetted to the different compartments of the grid (two compartments for each cell species). Constructs were placed in the incubator to allow cell adherence. (**F**) After 3 h of initial adherence, the constructs were transferred to 12-well tissue culture plates and were supplemented with osteocyte differentiation medium. (**G**) After 7 days of cultivation, the constructs were washed with PBS, gels were transferred to separate dishes for collagenase digest and membranes were cut with scissors. (**H**) Transwell insert with two membrane pieces seeded with osteoblasts (osteoclast-seeded membrane pieces were already transferred to separate dishes).

Osteocytes were differentiated in collagen gels, located in transwell inserts as described above. After 14 days of cultivation the medium was aspirated, the transwell inserts were placed bottom-up in 6-well plates and marked with a sterile surgical skin marker (Figure 8C). Silicon grids were placed on top of the transwell membranes (Figure 8D). Human osteoclasts were detached from their flasks as described above. Since osteoclasts are multinucleated cells, we refrained from counting them prior to seeding and used the suspension from one flask to seed 20 samples (consisting of two of the four compartments, formed by the silicon grids). Since PBMC containing 1 × 10^7^ monocytes were originally seeded to each flask, osteoclasts differentiated from 5 × 10^5^ monocytes were applied to each sample. Human osteoblasts, differentiated as described above and from the same donor as the osteocytes in the gel, were seeded to the other two compartments (5 × 10^4^ cells/sample). The cells were allowed to attach to the PET membrane without further medium supply for 3 h in the incubator. Subsequently, the silicon grids were removed, the constructs were transferred back into 12-well plates and osteocyte differentiation medium was added to the constructs as described above. Samples were cultivated for another 7 days without medium change. For experiments 6–9, a mixture of detached osteoclasts and osteoblasts was directly applied to the membranes of the transwell inserts.

Single cultures of osteocytes were performed in the same setup without adding osteoblasts and osteoclasts to the membranes of the transwell inserts. Single cultures of osteoblasts and osteoclasts were performed on the membranes of transwell inserts, filled with cell-free collagen gels.

Additional co-cultures of osteoblasts and osteoclasts were performed in the same setup: Transwell inserts filled with cell-free collagen gels were used and osteoblasts and osteoclasts were seeded, separated by silicon grids, onto the PET membrane. Cells of one osteoblast donor were combined with cells of two osteoclast donors in two independent experiments.

At the end of cultivation period, PET membranes were cut from the transwell inserts. For microscopic investigations, whole membranes were fixed with 4% formaldehyde in PBS. For all other investigations, membranes were cut into four parts and frozen separately until further analysis.

Osteocyte-containing gels were removed from the inserts and incubated with collagenase II solution (3 mg/mL collagenase II in α-MEM, 10% FCS, 2 mM L-glutamine, 100 U/mL penicillin and 100 µg/mL streptomycin, 3 mM CaCl2) for 1 h at 37 °C. The digests were transferred to 15 mL tubes, washed with PBS and centrifuged. The supernatant was discarded.

### 4.3. RNA Isolation, Preparation of cDNA and PCR

RNA was isolated from osteocyte-containing pellets which were obtained after collagenase digest of the collagen gels as well as from cut membrane pieces (experiments 1–5 only) using a commercially available kit (peqGOLD MicroSpin Total RNA Kit, Peqlab, Erlangen, Germany). Six samples of each group were used to generate three RNA samples, both for osteocytes, osteoblasts and osteoclasts. cDNA was obtained using the High-Capacity cDNA Reverse Transcription Kit (Applied Biosystems, Waltham, MA, USA) according to manufacturer’s instructions.

PCR reactions were set up using the TaqMan Fast Advanced Master Mix (Applied Biosciences, Beverly Hills, CA, USA) and TaqMan Gene Expression Assays for the following genes: glyceraldehyde-3-phosphate dehydrogenase (*GAPDH*), bone gamma-carboxyglutamate protein (osteocalcin, *BGLAP*), podoplanin (E11/g38; *PDPN*), phosphate regulating endopeptidase homolog, X-linked (*PHEX*), matrix extracellular phosphoglycoprotein (OPF 45, *MEPE*), receptor activator of NF-κB Ligand (RANKL, *TNFSF11*), alkaline phosphatase (*ALPL*), bone sialoprotein II (*BSP II*), tartrate-resistant acid phosphatase (*ACP5*), cathepsin K (*CTSK*) and carbonic anhydrase II (*CAII*) (Applied Biosystems), according to manufacturer’s instructions.

PCR was run with an Applied Biosystems^®^ 7500 fast Real-Time PCR system. Relative gene expression (fold-change) was calculated by using the 2−ΔΔCt method and normalized to single cultures of the respective cell species.

### 4.4. Analysis of Cell-Specific Enzyme Activities

Frozen membrane sections (experiments 1–5) were thawed and treated with 1% Triton X-100 in PBS for 50 min on ice. During lysis, a sonication step in ice water for 10 min was involved. Activities of ALP, TRAP, CAII and CTSK were quantified as previously described [57]. Briefly, ALP and CAII activity were determined using the cleavage of *p*-nitrophenyl phosphate to *p*-nitrophenol in different buffers and quantified by absorption measurement at 405 nm. TRAP activity was analyzed via the cleavage of Naphthol ASBI phosphate at acidic pH in the presence of tartrate, followed by fluorescence measurements at an excitation and emission wavelength of 405/520 nm. CTSK activity was evaluated due to the cleavage of Z-LR-AMC (Enzo Life sciences, Farmingdale, NY, USA) followed by the fluorescence measurement of free AMC at an excitation/emission wavelength of 365/440 nm.

### 4.5. Fluorescence Microscopy

Osteocyte-containing collagen gels and membranes, seeded with osteoblasts and osteoclasts (uncut) were fixed with 4% formaldehyde in PBS for 1 h at RT. After treatment with 0.1% Triton X-100 in PBS for 5 min the specimens were washed five times with PBS, followed by an incubation in 1 % BSA in PBS for 30 min (membranes) or 60 min (gels). Staining was performed using Alexa Fluor 488 phalloidin (Thermo Fisher Scientific) (5 U/mL) and (1 µg/mL) with an incubation time of 30 min (membranes) and overnight (collagen gels), respectively. Specimens were again washed with PBS and imaged using a Keyence BZ-X810 fluorescence microscope.

### 4.6. Statistics

For gene expression, analysis and enzyme activity measurements of osteoblasts and osteoclasts five different experiments were performed; gene expression of osteocytes was evaluated in nine independent experiments (Table 1). Statistical differences in gene expression of single and triple culture were calculated on the level of CT values as also reported elsewhere [19]. Data of the single experiments (n = 3) were compared using two-tailed students t-test and overall analysis of all five or nine experiments was performed using two-tailed Mann–Whitney analysis. * *p* < 0.05; ** *p* < 0.01, *** *p* < 0.001.

## 5. Conclusions

Human primary osteocytes, osteoclasts and osteoblasts can be combined in triple cultures in vitro, maintaining their typical morphology. Typical markers for osteoblasts, osteoclasts and osteocytes were expressed during triple culture of the three cell types and were detected on mRNA and, in some cases, on protein level. In osteoclasts, triple culture caused a downregulation of osteoclast-specific genes. In contrast, osteoblast markers ALP and BSPII were significantly upregulated in triple culture, which was mainly attributed to the stimulation by osteoclasts. The gene expression of the early osteocyte and late osteoblast marker osteocalcin was significantly downregulated in the triple cultures, which will be analyzed in more detail in future experiments. Since morphological features, as well as expression of typical markers, were evident in all examined triple culture experiments, those constructs might become a valuable tool to analyze the effect of biomaterial extracts, cytokines or bioactive ions on bone regeneration and turnover in vitro.

## Figures and Tables

**Table 1:** Donors and combination of cells for the different triple co-culture experiments.

Exp. No.	Osteoblasts (OB)/Osteocytes (Ocy)	Osteoclasts (OC)	Seeding OB/OC
1	male, 65 y	PBMC donor 1	patterned
2	male, 65 y	PBMC donor 2	patterned
3	female, 56 y	PBMC donor 1	patterned
4	female, 56 y	PBMC donor 3	patterned
5	female, 56 y	PBMC donor 4	patterned
6	female, 52 y	PBMC donor 5	mixed
7	female, 52 y	PBMC donor 6	mixed
8	female, 56 y	PBMC donor 5	mixed
9	female, 56 y	PBMC donor 6	mixed

## Data Availability

All data of this study is contained within the article.
